# Leave-one-out cross-validation, penalization, and differential bias of some prediction model performance measures—a simulation study

**DOI:** 10.1186/s41512-023-00146-0

**Published:** 2023-05-02

**Authors:** Angelika Geroldinger, Lara Lusa, Mariana Nold, Georg Heinze

**Affiliations:** 1 Center for Medical Data Science, Institute of Clinical Biometrics, Medical University of Vienna, Spitalgasse 23, 1090 Vienna, Austria; 2 Faculty of Mathematics, Natural Sciences and Information Technologies, University of Primorska, Koper, Slovenia; 3 Faculty of Medicine, University of Ljubljana, Ljubljana, Slovenia; 4 Department of Sociology, Friedrich Schiller University Jena, Jena, Germany

**Keywords:** Bootstrap, Concordance statistic, Discrimination slope, Logistic regression, Resampling techniques

## Abstract

**Background:**

The performance of models for binary outcomes can be described by measures such as the concordance statistic (c-statistic, area under the curve), the discrimination slope, or the Brier score. At internal validation, data resampling techniques, e.g., cross-validation, are frequently employed to correct for optimism in these model performance criteria. Especially with small samples or rare events, leave-one-out cross-validation is a popular choice.

**Methods:**

Using simulations and a real data example, we compared the effect of different resampling techniques on the estimation of c-statistics, discrimination slopes, and Brier scores for three estimators of logistic regression models, including the maximum likelihood and two maximum penalized likelihood estimators.

**Results:**

Our simulation study confirms earlier studies reporting that leave-one-out cross-validated c-statistics can be strongly biased towards zero. In addition, our study reveals that this bias is even more pronounced for model estimators shrinking estimated probabilities towards the observed event fraction, such as ridge regression. Leave-one-out cross-validation also provided pessimistic estimates of the discrimination slope but nearly unbiased estimates of the Brier score.

**Conclusions:**

We recommend to use leave-pair-out cross-validation, fivefold cross-validation with repetitions, the enhanced or the .632+ bootstrap to estimate c-statistics, and leave-pair-out or fivefold cross-validation to estimate discrimination slopes.

**Supplementary Information:**

The online version contains supplementary material available at 10.1186/s41512-023-00146-0.

## Introduction

The concordance statistic (c-statistic) is a widely used measure to quantify the discrimination ability of models for binary outcomes. Calculating the c-statistic for the data on which the model was fitted will usually give too optimistic results for the model performance in subjects outside of the model development set, especially with small samples or rare events. This over-optimism can be corrected by data resampling techniques such as cross-validation (CV) or the bootstrap. Leave-one-out (LOO) CV has the advantage of being applicable even with small samples where other techniques such as tenfold or fivefold CV might run into problems when, e.g., some of the CV subsets contain only one category of the binary outcome. Whereas LOO CV is known to yield nearly unbiased estimates for performance measures applicable to single observations such as the Brier score [16], it has been shown to induce negative bias into the c-statistic [1, 27]. Nevertheless, LOO CV to “cross-validate” estimated probabilities and c-statistics is still in widespread use. For example, in the current standard implementation of logistic regression in the SAS/STAT 15.2 and Viya 3.4 software, PROC LOGISTIC, a one-step approximation to LOO CV is the only built-in cross-validation method for estimated probabilities and also for the c-statistic [23].

The present paper aims to provide a better understanding of resampling techniques, in particular LOO CV, when combined with different estimators commonly used to fit logistic regression models with binary outcomes and when used to evaluate the model performance by means of the c-statistic, the Brier score, and the discrimination slope [30], which is an increasingly popular measure of predictive accuracy in binary outcome models. A similar aim was pursued by Iba et al. [15] but focusing on bootstrap methods only.

Throughout this paper, we focus on the situation of relatively few observations or events, where reliable predictive models are out of scope. Nevertheless, in such situations, it could still be of interest to use a prediction model to capture the data structure in a compact manner [26]. In such a model-based description of the association of an outcome with several covariates, an unbiased measure of the discrimination ability of that model is still of interest.

The remainder of this paper is organized as follows: we start with an introduction of the measures of model performance, the resampling techniques, and the model estimators of interest. A study on the association between diabetes and the waist-hip ratio serves as illustrative example. Subsequently, we provide an intuitive explanation of the problems with LOO CV using a simple toy example and present a comprehensive simulation study. Finally, we discuss the impact of our findings on routine statistical analyses. A preprint of this manuscript can be found on arXiv.org [9].

## Methods

### Measures of model performance

We denote the two outcome values as “event” and “non-event” and assume that in logistic regression, the probability of an event is modeled.

The c-statistic is the proportion of pairs among all possible pairs of observations with contrary outcomes in which the estimated event probability is higher in the observation with the event than in the observation with the non-event. It equals the area under the receiver operating characteristic curve [10].

The discrimination slope is the difference between the mean estimated probability of an event for observations with events and the mean estimated probability for observations with non-events. Paralleling the construction of the c-statistic, the discrimination slope can be computed as the average pairwise difference in estimated probabilities, thus representing a parametric version of the c-statistic. It was suggested as “highly recommendable *R*^2^-substitute for logistic regression models” by Tjur [30] and recently revisited by Antolini et al. [2]. For model estimators which give average estimated probabilities equal to the event fraction, the discrimination slope is equal to the measure of explained variation for binary outcomes proposed in [25]; see the Appendix for a proof.

The Brier score is the mean squared difference between the binary outcome and the estimated probabilities [29]. It equals 0 for perfect models. The magnitude of the Brier score has to be interpreted in the context of the event fraction of a data set. For instance, at an event fraction of 0.5, a non-informative model with estimated probabilities equal to the event fraction assumes a Brier score of 0.25, while at an event fraction of 0.25, it is 0.1875. Unlike the c-statistic and the discrimination slope, the Brier score can be defined and computed for single observations.

### Techniques to correct for over-optimism

Throughout this paper, we assume that the observations in the model development set are drawn from a large underlying population and that we are interested in the performance of the model applied to this underlying population excluding the observations in the development set. Calculating performance measures on the same data as used for model development will usually result in highly biased (“over-optimistic”) estimates which need to be corrected. Here, we describe some resampling techniques which provide optimism-corrected estimates of the c-statistic. If not mentioned otherwise, methodology straightforwardly generalizes to the discrimination slope and the Brier score. We denote by “apparent” measures that are calculated from the data on which the model was fitted without correction.

With *f*-fold CV, the data are split into *f* parts or “folds” with approximately equal number of observations. A model is then fitted on the observations from *f*-1 folds. Using this model, estimated probabilities for the observations in the excluded *f*th fold are calculated. By excluding each fold in turn, one obtains *f* c-statistics which are then averaged. To decrease variability caused by the random partitioning, the whole procedure is repeated *r* times and results are averaged. Here, we consider *f* = 5 and *r* = 40, i.e., 5-fold CV with 40 repetitions. For brevity, we will not always explicitly mention these repetitions, but we always performed repeated 5-fold CV.

Setting *f* to the sample size *n* (“leave-one-out CV”), the c-statistic cannot be computed using the averaging strategy explained above, as only one observation is excluded at each iteration. Instead, one has to resort to a pooling strategy by fitting *n* models using each possible subset of *n*-1 observations, each time calculating the predictive probability for the observation excluded from model estimation, and computing a single c-statistic from the pooled *n* estimated probabilities. Notably, for performance measures applicable to single observations such as the Brier score, LOO CV can also be applied in conjunction with the averaging strategy and then gives the same result as the pooling strategy.

Leave-pair-out (LPO) CV is an approach that is independent of random sampling but based on c-statistics calculated within folds [1, 27]. With LPO CV, each pair of observations with contrary outcomes is excluded from the data and, in turn, a model is fitted on the remaining *n* − 2 observations, and estimated probabilities for the two excluded observations are calculated from this model. The LPO cross-validated c-statistic is the proportion of pairs with concordant estimated probabilities, i.e., where the estimated probability of the observation with the event is higher than that of the observation with the non-event. LPO CV can imply considerable computational burden: if *k* is the number of events, (*n* − *k*)*k* models have to be estimated, compared to only *n* models with LOO CV. For example, with 50 events among 100 observations, 2500 models must be fitted with LPO but only 100 with LOO CV. Whereas LPO CV generalizes straightforwardly to the discrimination slope, it is not clear how it should be adapted for the Brier score: simply averaging the Brier score computed for all left-out pairs will give biased estimates in the case of unbalanced outcomes because of the dependence of the Brier score on the event fraction. One solution would be to adequately weight contributions by events and non-events. Here, we refrain from applying LPO CV to the Brier score.

In Harrell’s implementation of an enhanced bootstrap to correct bias due to overfitting [12], the bias is explicitly estimated and then subtracted from the apparent c-statistic. Specifically, 200 samples of *n* observations with replacement are drawn from the original data set. On each of these bootstrap resamples, a model is fitted and used to calculate c-statistics both for the bootstrap resample and the original data. An estimate of “optimism” is obtained by subtracting the average c-statistic in the original data from the average c-statistic in the bootstrap resamples. The enhanced bootstrap c-statistic is then given by the apparent c-statistic minus the estimate of optimism.

The .632+ bootstrap [7] is a weighted average of the apparent c-statistic and the average “out-of-the-bag” c-statistic calculated from bootstrap resamples. The “out-of-the-bag” c-statistic is obtained by fitting the model in a bootstrap resample and applying it to the observations not contained in that bootstrap resample. We give the technical details in the Appendix.

### Penalized likelihood estimation methods

We investigated the performance of the resampling techniques in combination with the following estimators of logistic regression:

- Maximum likelihood estimation (ML)
- Firth’s penalized logistic regression (FL) [8, 14]
- Logistic ridge regression (RR) [18]

FL amounts to penalization by the Jeffreys prior and was shown to reduce the bias in coefficient estimates compared to ML. With RR, the log likelihood is penalized by the square of the Euclidean norm of the regression parameters multiplied by a tuning parameter. We chose the tuning parameter by minimizing a penalized version of the Akaike’s information criterion (AIC) given by $-2l\left(\hat{\beta}\right)+2\ d{f}_e$ with $l\left(\hat{\beta}\right)$ the log likelihood,

$$df_e=\text{trace}\left(\frac{\partial^2l}{\partial\beta^2}\left(\widehat\beta\right)\left(\frac{\partial^2l^\ast}{\partial\beta^2}\left(\widehat\beta\right)\right)^{-1}\right)$$

the effective degrees of freedom, and ${l}^{\ast}\left(\hat{\beta}\right)$ the penalized log likelihood [33]. This approach of optimizing the tuning parameter is less computer-intensive than the optimization of cross-validated measures and has been reported to yield similar or even superior results [12].

For the implementation of ML and FL, we used the R-package logistf with default convergence criteria [13, 22]. For RR, we applied the function lrm in the R-package rms, setting the singularity criterion to 10^−15^ [11].

If the data are separated, i.e., if a combination of explanatory variables perfectly predicts the outcome, then ML fails to produce finite regression coefficients, and some estimated probabilities will be exactly 0 or 1 [19]. By contrast, FL gives reasonable results in the case of separation. Under separation, RR will supply finite regression coefficients if the tuning parameter is greater than 0. However, CV or AIC optimization will often set the tuning parameter to 0 in case of separation, and then RR leads to the same problems as ML [19]. See Additional file 1: S1 for how we handled separation, linearly dependent explanatory variables, or binary outcomes restricted to one category occurring in bootstrap resamples or CV subsets.

## Motivation

### A real data example: association between waist-hip ratio and diabetes

**Fig. 1:**
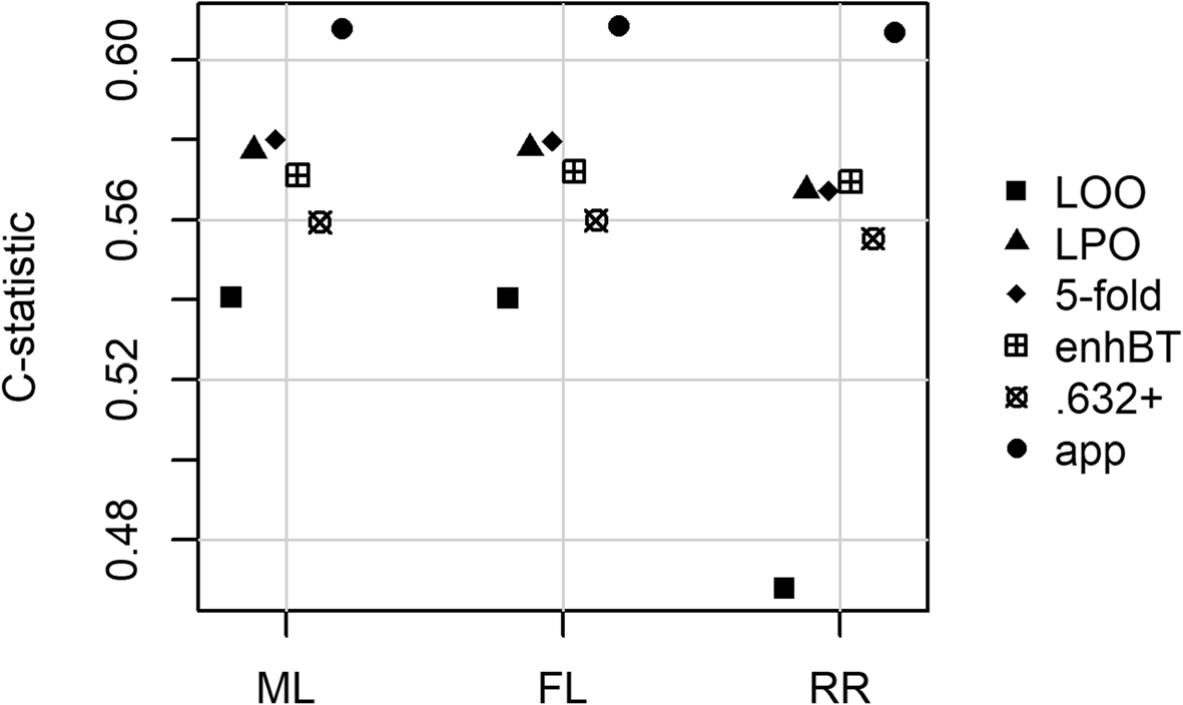
Apparent and optimism-corrected c-statistics for three different estimators of logistic regression models using data from a screening examination on coronary heart disease risk factors. ML, maximum likelihood; FL, Firth’s logistic regression; RR, ridge regression. LOO, leave-one-out cross-validation; LPO, leave-pair-out cross-validation; 5-fold, 5-fold cross-validation; enhBT, enhanced bootstrap; .632+, .632+ bootstrap; app, apparent estimate

As part of a smoking cessation project among Afro-Americans in two rural Virginia counties, a screening examination on coronary heart disease risk factors was performed [34]. For illustration purposes, we focus on the association between the waist-hip ratio and the presence of diabetes (defined by glycosylated hemoglobin >7.0), adjusted for gender, in the Virginia county Louisa. Among the 198 study participants, 14.6% (29 persons) were classified as having diabetes. A difference of 0.10 in the waist-hip ratio was associated with an adjusted odds ratio, estimated with ML, of 1.9 (95% CI; 1.01, 3.58). On the level of estimated probabilities, this corresponds to probabilities of diabetes of 0.112 and 0.193 for females with a waist-hip ratio of 0.8 or 0.9, respectively. In line with findings from previous studies [1, 27], LOO CV resulted in lower c-statistics for ML, FL, and RR than the other resampling techniques; see Fig. 1. While a model with no discriminative ability (a “random guess”) would yield a c-statistic of approximately 0.5, the LOO cross-validated c-statistic for RR was even only 0.468, while the corresponding c-statistics of ML and FL both were 0.54. This may give the impression that RR supplies a model that performs even worse than a random guess, while ML and FL yield better models. All other resampling techniques gave similar c-statistics across model estimators.

### Understanding the bias in LOO cross-validated c-statistics

**Fig. 2:**
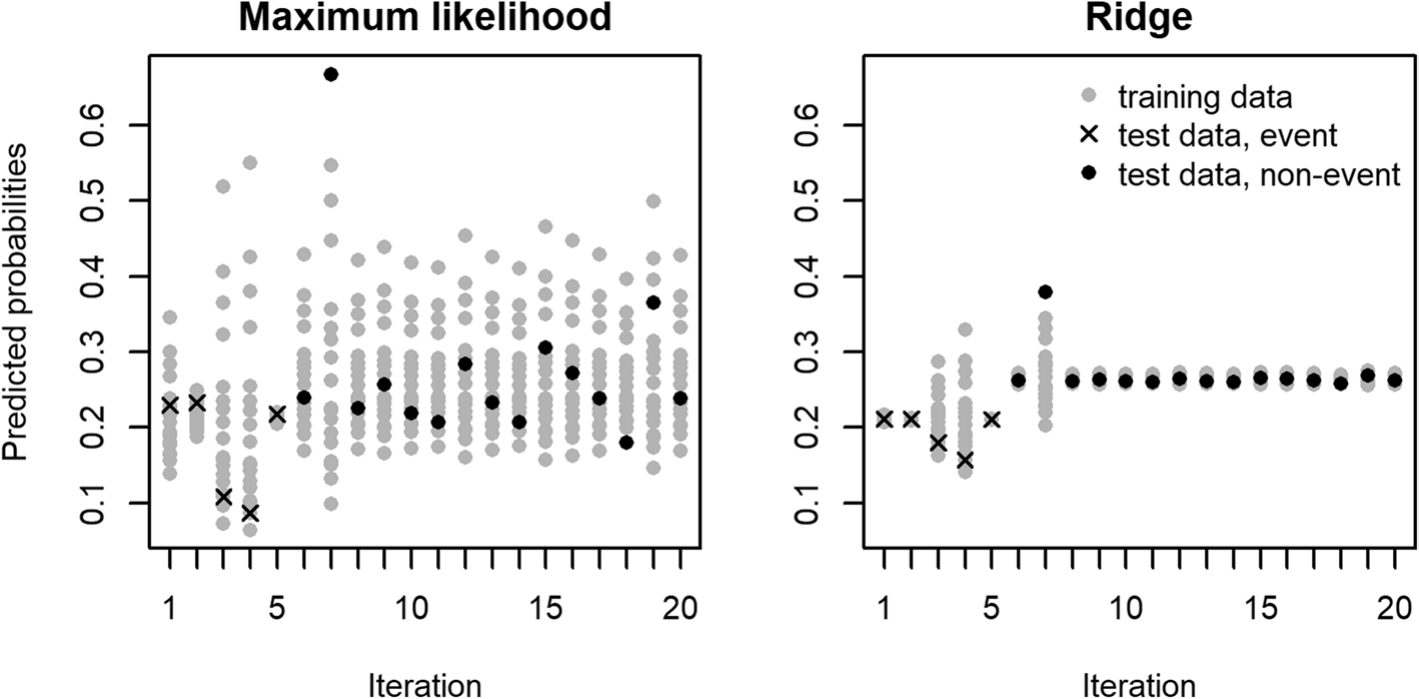
Illustration of the calculation of the c-statistic in leave-one-out cross-validation for the maximum likelihood and ridge estimators. Data consisted of 20 observations of a normally distributed explanatory variable, with 5 randomly chosen observations labeled as “events” (*t*-test *p*-value = 0.584). Each tick on the *x*-axis corresponds to one of the 20 iterations in leave-one-out cross-validation. Grey symbols mark estimated probabilities for the 19 observations used in the model fitting; black symbols mark the estimated probabilities for the left-out observations. Black crosses or circles indicate that the left-out observation corresponds to an event or non-event, respectively. The leave-one-out cross-validated c-statistic was equal to 0.17 for maximum likelihood estimation and equal to 0 for ridge regression

Figure 2 explains the bias in LOO cross-validated c-statistics by illustrating the estimation process on an artificial toy example with 20 observations and 5 events. The crucial observation in Fig. 2 is that the estimated probability for a left-out event (CV iterations 1–5) was on average lower than for a left-out non-event (CV iterations 6–20). If an event was left out, the data used in the model fitting consisted of only 4 events out of 19 observations (event fraction 0.210), compared to 5 out of 19 (event fraction 0.263) if a non-event was left out. Hence, the LOO cross-validated estimated probability tends to be too low for an event and too high for a non-event. Consequently, LOO cross-validated c-statistics (and discrimination slopes) based on pooling these cross-validated estimated probabilities are biased low. Figure 2 also illustrates that the bias in LOO cross-validated c-statistics is usually more severe for modeling methods yielding shrunken estimated probabilities such as ridge regression. This tendency can lead to undesired results if one optimizes the tuning parameter in RR using LOO cross-validated c-statistics; see Additional file 1: Figure S1. Whereas for the null scenario, the discrimination ability of RR is independent of the penalization strength, optimization of LOO cross-validated c-statistics favors models with less regularization.

## Simulation study

We follow the ADEMP structured approach in describing the setup of our simulation study [21].

### Aim

The aim of the simulation study was to compare the accuracy of the resampling techniques LOO CV, LPO CV, 5-fold CV, enhanced bootstrap, and .632+ bootstrap in estimating c-statistics, discrimination slopes, and Brier scores for the model estimators ML, FL, and RR. In particular, the following questions should be answered:

- Is the discrimination slope accurately estimated by LOO CV?
- Does the performance of resampling methods differ between model estimators?
- Which resampling methods estimate the c-statistic, the discrimination slope, and the Brier score most efficiently?

### Data generating mechanism

Data generation was motivated by the structure of real data sets, where typically a mix of variables with different distributions is encountered [5]. By sampling from a multivariate normal distribution and applying certain transformations, we generated one binary, one ordinal, and three continuous explanatory variables; see Additional file 1: S2 and Table S1 for details. Binary outcomes *y*_*i*_ were drawn from Bernoulli distributions with the event probability following a logistic model. We considered twelve simulation scenarios in a factorial design combining sample size (*n* ∈ {50,100}), marginal event fraction (*E*(*y*) ∈ {0.25, 0.5}), and effect size (strong or weak effects of all explanatory variables, or null scenarios with no effects). More information on the magnitude of the effects is given in Additional file 1: S2. For each scenario, we created 1000 data sets.

### Estimands

Our estimands are the c-statistic, the discrimination slope, and the Brier score for the model estimators ML, FL, and RR.

### Methods

For each simulated dataset and each model estimator, we assessed the predictive accuracy in terms of c-statistics, discrimination slopes, and Brier scores by the five resampling methods LOO CV, LPO CV, 5-fold CV, enhanced bootstrap, and .632+ bootstrap.

### Performance measures

We compared the resampling-based c-statistics, discrimination slopes, and Brier scores with those obtained if the estimated models were validated in the population, in our study approximated by an independent validation data set consisting of 100,000 observations. We described the performance of the resampling techniques in terms of mean and root mean squared difference (RMSD) of the resampling-based c-statistics, discrimination slopes, and Brier scores to their respective independently validated (IV) counterparts. Finally, we calculated Monte Carlo standard errors for the mean squared difference [21] and the RMSD [17].

### Results

First, we describe the distribution of the c-statistic, discrimination slope, and Brier score obtained in the independent validation set, which will serve as gold standard. The mean IV c-statistics ranged between 0.5 and 0.684; see Additional file 1: Table S2. RR achieved the largest mean IV c-statistics in non-null scenarios, but there was little difference between model estimators.

For the mean IV discrimination slope, the differences between the model estimators showed a range of up to 0.04 units; see Additional file 1: Table S3. In non-null scenarios, ML achieved the largest median IV discrimination slopes, with values of up to 0.135. RR yielded the smallest median IV discrimination slopes, which were at least 20% smaller than by ML in all scenarios.

The results for the IV Brier score were in contrast to those for the IV discrimination slope: now, ML performed worst in all scenarios, while RR resulted in the smallest mean Brier scores in all but one scenarios; see Additional file 1: Table S4.

**Fig. 3:**
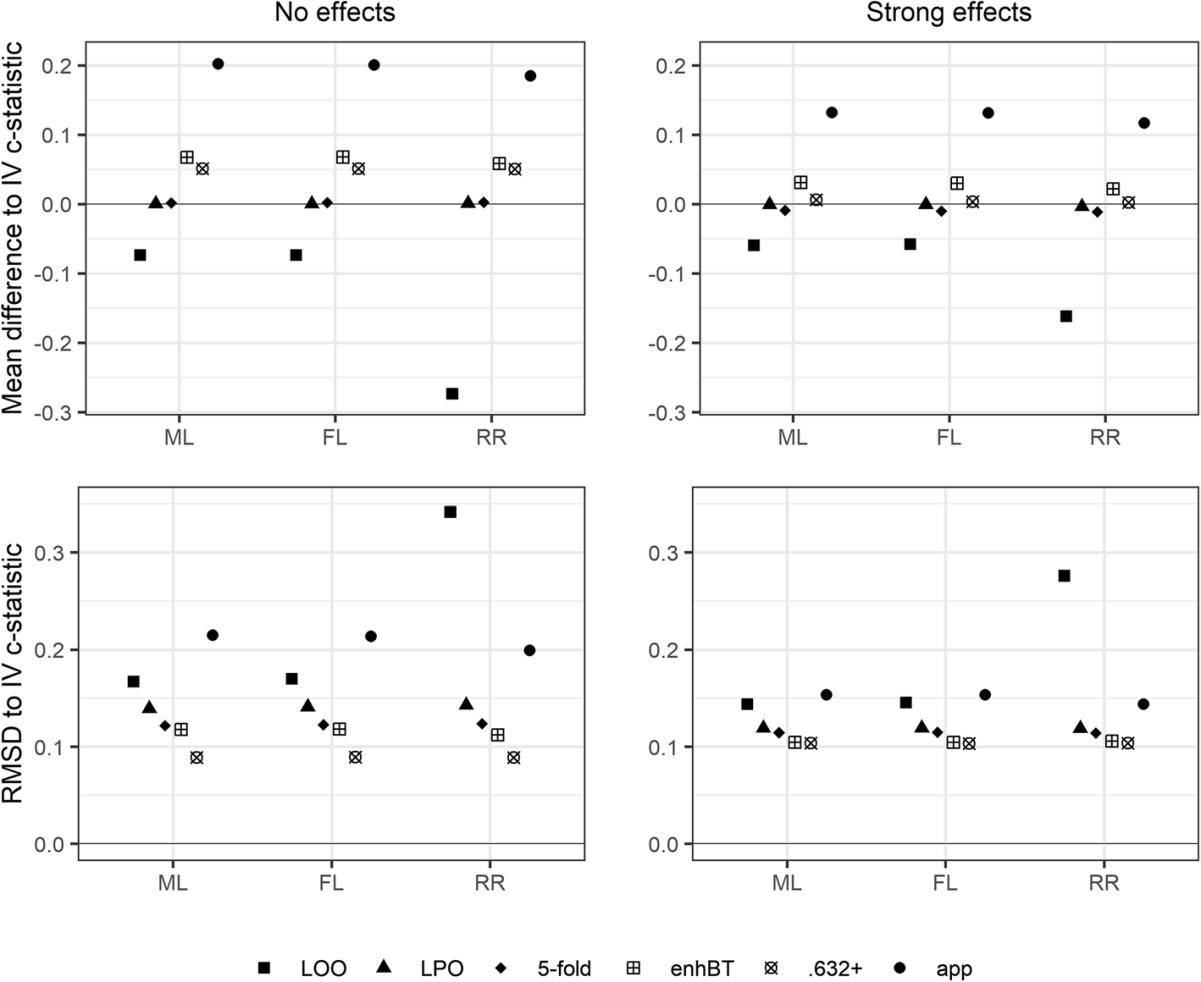
Mean differences and root mean squared differences (RMSD) between c-statistics computed by data resampling techniques and the independently validated (IV) c-statistic for the model estimators ML, FL, and RR for the simulation settings with 50 observations, an event fraction of 0.25, and either no or strong effects. The Monte Carlo standard errors of the mean difference and of the root mean squared difference were smaller than 0.008 and 0.007, respectively, for all scenarios. ML, maximum likelihood; FL, Firth’s logistic regression; RR, ridge regression. LOO, leave-one-out cross-validation; LPO, leave-pair-out cross-validation; 5-fold, 5-fold cross-validation; enhBT, enhanced bootstrap; .632+, .632+ bootstrap; app, apparent estimate

In approximating IV c-statistics, LOO CV performed worst both with respect to mean difference (bias) and RMSD; see Fig. 3. The downward bias was most severe for RR and amounted to − 0.274 in the most unfavorable scenario. For this scenario, the magnitude of the bias with ML or FL was only about a quarter of the magnitude of the bias with RR. In all but two scenarios, the enhanced and the .632+ bootstraps yielded the smallest RMSD for RR, ML, and FL. Notably, the RMSD increased with increasing effect size for the .632+ bootstrap, whereas it decreased for all other resampling methods as expected. This behavior can be understood by looking at the definition of the .632+ bootstrap, which ensures that the .632+ c-statistic is always greater than or equal to the minimum of the apparent c-statistic and 0.5, resulting in a right-skewed distribution of the .632+ c-statistic especially for null scenarios. For all model estimators and all resampling techniques, the RMSD decreased with increasing sample size and increasing event fraction. The differences between resampling techniques were less pronounced with stronger effects, larger sample sizes, and balanced event fraction.

**Fig. 4:**
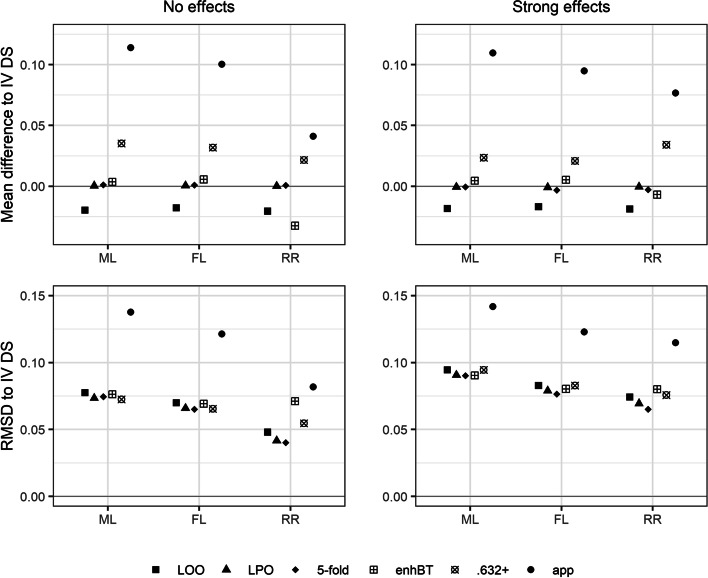
Mean differences and root mean squared differences (RMSD) between discrimination slopes (DS) computed by data resampling techniques and the independently validated (IV) DS for the model estimators ML, FL, and RR for the simulation settings with 50 observations, an event fraction of 0.25, and either no or strong effects. The Monte Carlo standard errors of the mean difference and of the root mean squared difference were smaller than 0.003 and 0.007, respectively, for all scenarios. ML, maximum likelihood; FL, Firth’s logistic regression; RR, ridge regression. LOO, leave-one-out cross-validation; LPO, leave-pair-out cross-validation; 5-fold, 5-fold cross-validation; enhBT, enhanced bootstrap; .632+, .632+ bootstrap; app, apparent estimate

LOO CV also performed poorly in approximating the IV discrimination slope, yielding pessimistic estimates with a RMSD at least larger than the one by LPO CV and 5-fold CV; see Fig. 4. However, the differences in RMSD across resampling techniques were fairly small. Only for RR the two bootstrap techniques sometimes resulted in discrimination slopes with substantially larger RMSD than LPO CV and 5-fold CV. The .632+ bootstrap gave overly optimistic discrimination slopes, with an absolute mean difference to the IV values often larger than the one by LOO CV. On the other hand, in all but three simulation scenarios, the .632+ bootstrap yielded discrimination slopes with smallest median deviations. This discrepancy can be explained by the right-skewness of the distribution of the differences between optimism-corrected and IV discrimination slopes, which was especially pronounced for the .632+ bootstrap. With increasing sample size, the RMSD decreased for all resampling techniques and all model estimators. Again, the differences between resampling techniques were less pronounced with increasing effect size, sample size, and more balanced event fraction.

As described in the “Methods” section, LPO CV does not naturally generalize to the Brier score, so we only considered LOO CV, 5-fold CV, enhanced bootstrap, and .632+ bootstrap. In all but three simulation scenarios, LOO CV performed best with respect to the mean difference to the IV Brier score for all model estimators; see Additional file 1: Figure S2. Similarly, as for the discrimination slope, the enhanced bootstrap gave overly pessimistic Brier scores with fairly large RMSD for RR, especially in scenarios with no or small effects. However, differences in RMSD were small between resampling methods, only in scenarios with small or no effects the .632+ bootstrap showed some benefit over the other techniques. With increasing sample size, the RMSD decreased for all resampling techniques and all model estimators.

The percentage of separated data sets was highest (18.2%) for the scenario with a sample size of 50, an event fraction of 0.25, and strong effects; see Additional file 1: Table S5. In this scenario, more than one third of bootstrap resamples were separated.

## Discussion

The findings of our simulation study confirm that LOO CV yields pessimistic c-statistics [1, 27], but they also demonstrate that this bias depends on the choice of model estimator. Thus, LOO cross-validated c-statistics should neither be used to describe the performance of a single model nor to compare the performance of a set of models estimators, e.g., in the optimization of tuning parameters in regularized regression. LPO CV, which was suggested as an alternative to LOO CV [1], indeed performed better both in terms of mean difference and RMSD to the IV c-statistic. However, the enhanced bootstrap and the .632+ bootstrap achieved a smaller RMSD in almost all simulation settings. The .632+ bootstrap is a weighted average of the apparent c-statistic and a certain overly corrected c-statistic which is set to 0.5 if smaller. In this way, it is ensured that the .632+ bootstrap c-statistics are greater than or equal to 0.5 (or the apparent c-statistic if the apparent c-statistic should be smaller than 0.5). One can apply a similar kind of winsorization with any resampling technique by reporting c-statistics smaller than 0.5 as 0.5, whereas in practice winsorizing at 0.5 rather entails a loss of information instead of a gain in precision, this approach leads to smaller RMSD to the IV c-statistic in simulations; see Additional file 1: Table S6. With this in mind, the superiority of the .632+ bootstrap in terms of RMSD to the IV c-statistic might appear less relevant. The performance of LPO CV, 5-fold CV, enhanced bootstrap, and .632+ bootstrap in the estimation of c-statistics was too similar to give definite recommendations in favor of one of these techniques, which is in line with a previous study [27]. Thus, the choice might be guided by other criteria such as the dependency on data sampling, the extent of computational burden, the level of complexity of the approach, or the likeliness of encountering problems with model fitting in resamples. In particular, if in addition to the c-statistic the corresponding receiver operating characteristics curve should be estimated, different methods are required [20], since the resampling techniques discussed above do not provide rankings of the data necessary for estimating receiver-operating characteristic curves. One important point left aside in our simulation study is that if one wants to accurately estimate the performance of a model estimator consisting of multiple steps, e.g., a variable selection step and a coefficient estimation step, the model development should be systematically replayed in every bootstrap or CV sample, as emphasized by [28].

LOO CV also gives pessimistic estimates for discrimination slopes. Moreover, our simulations revealed unexpected behavior of some of the bootstrap techniques. First, the enhanced bootstrap and the .632+ bootstrap performed reasonably well for ML and FL but sometimes poorly for RR in estimating the discrimination slope. Second, the simple bootstrap resulted in estimates even more optimistic than the apparent discrimination slopes; see Additional file 1: S3. According to our simulation results, we suggest using LPO CV or 5-fold CV to correct for optimism in discrimination slopes.

The Brier score is the only performance measure considered in this study which can be estimated by LOO CV using the averaging approach. As expected from the general theory on LOO CV [16], the LOO cross-validated Brier scores were close to unbiased in our simulation. For the Brier score, there was little difference between resampling methods.

According to Austin and Steyerberg [3], the split-sample method, where a proportion of the data is excluded from model fitting and later used as independent data set for assessing the model performance, is quite popular among clinical investigators. However, we have not included the split-sample method in our simulation study as it is known to perform poorly, resulting in estimates of even larger RMSE than the apparent estimates [3]. Various authors proposed to use the internally leave-one-out cross-validated calibration slope as global shrinkage factor to correct predictions for overestimation [6, 24, 31, 32]. We plan to investigate the calibration slope in this context in a separate report. Another interesting topic of future research is the reliable estimation of variability for internally validated performance measures (see, e.g., [4]).

Our study illustrates that the performance of resampling techniques can vary considerably between model estimators, even if the model estimators are similar in construction. Including for instance machine learning methods such as support vector machines into the comparison might even have revealed larger performance differences. This interaction between resampling techniques and model estimators implies that simulation studies aiming to assess the accuracy of a resampling technique should consider a broader set of model estimators to be widely applicable.

Summarizing, our study emphasizes that estimates provided by resampling techniques should be treated with caution, no matter whether one is interested in absolute values or a comparison between model estimators. Especially in studies with small samples or possibly spurious effects, it might be reasonable to scrutinize the validity of a performance measure estimate by applying an alternative resampling method.

### Supplementary Information

**Additional file 1: **S1. Problems in resampling techniques associated with small samples. S2. Data generating mechanism. S3. A side remark on the simple bootstrap: resampling may increase the optimism. **Figure S1.** Independently validated (solid line) and leave-one-out crossvalidated (dashed line) c-statistics for different penalization strengths in ridge regression on six artificially constructed data sets. The data were created in the same way as for one of the scenarios in our simulation study (null scenario, sample size of 50, marginal event fraction of 0.25). The x-axis shows the tuning parameter in ridge regression (lambda in the R package glmnet) with higher values corresponding to stronger penalization. For each data set we fitted 96 ridge regression models corresponding to a series of log-equidistant tuning values. As in our simulation study, the independently validated c-statistics were obtained by validating the models on an independent data set consisting of 100,000 observations. As expected, the independently validated c-statistics are very close to the true value of 0.5. LOO, leave-one-out crossvalidation; IV, independently validated. **Figure S2.** Mean and root mean squared differences (RMSD) between Brier scores (BS) computed by data resampling techniques and independently validated (IV) BS for three different model estimators for the simulation settings with 50 observations, an event fraction of 0.25 and either no or strong effects. The Monte Carlo standard errors of both, the mean difference and of the root mean squared difference (x100), were smaller than 0.2 for all scenarios. ML, maximum likelihood; FL, Firth’s logistic regression; RR, ridge regression. LOO, leave-one-out crossvalidation; 5-fold, 5-fold crossvalidation; enhBT, enhanced bootstrap; .632+, .632+ bootstrap; app, apparent estimate. **Table S1.** Construction of explanatory variables in the simulation study, following Binder H, Sauerbrei W, Royston P. Multivariable Model-Building with Continuous Covariates: 1. Performance Measures and Simulation Design. Germany: University of Freiburg; 2011. Square brackets […] indicate that the argument is truncated to the next integer towards 0. The indicator function 1_{…}_ is equal to 1 if the argument is true and 0 otherwise. **Table S2.** Mean and standard deviation (x100) of independently validated (IV) c-statistics for different model estimators and all simulation scenarios. The standard deviation strongly depends on the number of new observations (in our case 100 000) used to estimate the IV c-statistics. **Table S3.** Mean and standard deviation (x100) of independently validated (IV) discrimination slope for different model estimators and all simulation scenarios. The standard deviation strongly depends on the number of new observations (in our case 100 000) used to estimate the IV discrimination slope. **Table S4.** Mean and standard deviation (x100) of independently validated (IV) Brier score for different model estimators and all simulation scenarios. The standard deviation strongly depends on the number of new observations (in our case 100 000) used to estimate the IV Brier score. **Table S5.** Percentage of separated data sets for the twelve simulation scenarios in the full data sets, in the data sets used for model fitting in leave-one-outcrossvalidation, leave-pair-out crossvalidation and 5-fold crossvalidation, respectively, and in the bootstrap data sets. **Table S6.** Mean difference and root mean squared difference (x100) between winsorized c-statistics computed by different resampling techniques and the independently validated (IV) value (as presented in Table S2) for simulation scenarios with sample size of 50 and event fraction of 0.25. Resampled c-statistics were winsorized by replacing values smaller than 0.5 by 0.5. Figure 3 shows the analogous results for the untransformed c-statistics.

## Data Availability

The data on the screening examination as part of the smoking cessation project is freely available at http://biostat.mc.vanderbilt.edu/DataSets.

## Appendix

### Reinterpreting the discrimination slope

Here, we show that for an arbitrary model estimator which gives estimated probabilities ${\hat{\pi}}_i$ which are on average equal to the mean $\overline{y}$ of the binary outcome *y* = (*y*_*i*_), *y*_*i*_ ∈ {0, 1}, *i* = 1, …*n*, the discrimination slope is equal to the measure of explained variation ${\hat{V}}_B$ defined by Schemper [25]. Denote by $\overline{\pi}={n}^{-1}\left({\sum}_i{\hat{\pi}}_i\right)$ the average estimated probability, by assumption, we have $\overline{\pi}=\overline{y}$. The explained variation ${\hat{V}}_B$ is defined as

$${\hat{V}}_B=\frac{\left({n}^{-1}\sum \left|{y}_i-\overline{\pi}\right|-{n}^{-1}\sum \left|{y}_i-{\hat{\pi}}_i\right|\right)}{n^{-1}\sum \left|{y}_i-\overline{\pi}\right|}.$$

The denominator is equal to $2\overline{\pi}\left(1-\overline{\pi}\right)$ as can be verified by splitting the sum into summands with *y*_*i*_ = 0 and summands with *y*_*i*_ = 1, noting that there are $n\left(1-\overline{\pi}\right)$summands of the first type and $n\overline{\pi}$summands of the second type. Similarly, we can decompose

$$\sum\left|y_i-{\widehat\pi}_i\right|=n\overline\pi-\sum\nolimits_{i:y_i=1}{\widehat\pi}_i+\sum\nolimits_{i:y_i=0}{\widehat\pi}_i$$

$$=2\sum_{i:y_i=0}{\widehat\pi}_i,$$

where we have used that ${\sum}_{i:{y}_i=1}{\hat{\pi}}_i$ is equal to $n\overline{\pi}-{\sum}_{i:{y}_i=0}{\hat{\pi}}_i$. Summarizing, ${\hat{V}}_B$ can be calculated as

$${\hat{V}}_B=\frac{n\overline{\pi}\left(1-\overline{\pi}\right)-{\sum}_{i:{y}_i=0}{\hat{\pi}}_i}{n\overline{\pi}\left(1-\overline{\pi}\right)}.$$

Using the notation introduced above, the discrimination slope can be computed as $\frac{\sum_{i:{y}_i=1}{\hat{\pi}}_i}{n\ \overline{\pi}}-\frac{\sum_{i:{y}_i=0}{\hat{\pi}}_i}{n\ \left(1-\overline{\pi}\right)}.$ Again exploiting that ${\sum}_{i:{y}_i=1}{\hat{\pi}}_i=n\overline{\pi}-{\sum}_{i:{y}_i=0}{\hat{\pi}}_i$, we conclude that the discrimination slope and the measure of explained variation ${\hat{V}}_B$ agree.

### Applying the .632+ bootstrap to the c-statistic

The .632+ bootstrap was introduced as a tool providing optimism corrected estimates for error rates [7]. It allows for different choices of the particular form of this error rate but assumes that the error rate can be assessed on the level of observations, i.e., quantifies the discrepancy between a predicted value and the corresponding observed outcome value. As both the c-statistic and the discrimination slope cannot be applied to single observations but only to collections of observations, we had to slightly modify the definitions.

The .632+ bootstrap estimate of the c-statistic, ${\hat{c}}^{.632+},$ is a weighted average of the apparent c-statistic ${\hat{c}}^{\textrm{app}}$ and an overly corrected bootstrap estimate ${\hat{c}}^{(1)}$. It is constructed as follows: the model is fitted on each of, say 200 bootstrap resamples (i.e., random samples of size *n* drawn with replacement), and is used to calculate the estimated probabilities for the observations omitted from the bootstrap resample. For each of the bootstrap resamples, the c-statistic is then calculated from the omitted observations. Finally, these c-statistics are averaged over all bootstrap resamples yielding the estimate ${\hat{c}}^{(1)}$.

The .632+ bootstrap estimate of the c-statistic is then given by

$${\hat{c}}^{.632+}=\left(1-\hat{w}\right)\cdot {\hat{c}}^{\textrm{app}}+\hat{w}\cdot {\hat{c}}^{(1)},$$

where $\hat{w}=0.632/\left(1-0.368\ \hat{R}\right)$ with $\hat{R}=\left({\hat{c}}^{\textrm{app}}-{\hat{c}}^{(1)}\right)/\left(\ {\hat{c}}^{\textrm{app}}-0.5\right).$ In order to ensure that $\hat{R}$ falls between 0 and 1 such that $\hat{w}$ ranges from 0.632 to 1, the following modifications are made:

- set ${\hat{c}}^{(1)}$ to 0.5 if ${\hat{c}}^{(1)}$ is smaller than 0.5 and

- set $\hat{R}$ to 0 if ${\hat{c}}^{(1)}$> ${\hat{c}}^{\textrm{app}}$ or if $0.5\ge {\hat{c}}^{\textrm{app}}$.

The value 0.5 occurring in these modifications and in the denominator of $\hat{R}$ is the expected c-index if the outcome is independent of the explanatory variables. The .632+ bootstrap estimate of the discrimination slope can be obtained analogously, just replacing 0.5 by 0 in the definitions above.
