# Supplementary material for: Leave-one-out cross-validation, penalization, and differential bias of some prediction model performance measures—a simulation study
Source: Diagn Progn Res. 2023 May 2;7:9. doi: 10.1186/s41512-023-00146-0 (PMC10152625; doi:10.1186/s41512-023-00146-0)
Supplement: Supplementary file 1 — Additional file 1: S1. Problems in resampling techniques associated with small samples. S2. Data generating mechanism. S3. A side remark on the simple bootstrap: resampling may increase the optimism. Figure S1. Independently validated (solid line) and leave-one-out crossvalidated (dashed line) c-statistics for different penalization strengths in ridge regression on six artificially constructed data sets. The data were created in the same way as for one of the scenarios in our simulation study (null scenario, sample size of 50, marginal event fraction of 0.25). The x-axis shows the tuning parameter in ridge regression (lambda in the R package glmnet) with higher values corresponding to stronger penalization. For each data set we fitted 96 ridge regression models corresponding to a series of log-equidistant tuning values. As in our simulation study, the independently validated c-statistics were obtained by validating the models on an independent data set consisting of 100,000 observations. As expected, the independently validated c-statistics are very close to the true value of 0.5. LOO, leave-one-out crossvalidation; IV, independently validated. Figure S2. Mean and root mean squared differences (RMSD) between Brier scores (BS) computed by data resampling techniques and independently validated (IV) BS for three different model estimators for the simulation settings with 50 observations, an event fraction of 0.25 and either no or strong effects. The Monte Carlo standard errors of both, the mean difference and of the root mean squared difference (x100), were smaller than 0.2 for all scenarios. ML, maximum likelihood; FL, Firth’s logistic regression; RR, ridge regression. LOO, leave-one-out crossvalidation; 5-fold, 5-fold crossvalidation; enhBT, enhanced bootstrap; .632+, .632+ bootstrap; app, apparent estimate. Table S1. Construction of explanatory variables in the simulation study, following Binder H, Sauerbrei W, Royston P. Multivariable Model-Building with Continuous [file 41512_2023_146_MOESM1_ESM.docx]

Supplement for

“Leave-one-out cross-validation, penalization and differential bias of some prediction model performance measures – a simulation study”

by Angelika Geroldinger, Lara Lusa, Mariana Nold, and Georg Heinze

S1. Problems in resampling techniques associated with small samples

With small samples one frequently encounters separation in bootstrap resamples or CV subsets even if the original data are not separated. This can lead to problems with methods not being capable of dealing with separated data such as ML or RR. In this study, we decided to follow the simple strategy of restricting the number of iterations in the estimation process and using the results from the last iteration even if ML and RR did not converge due to separation. A more sophisticated strategy would be to replace the model estimation method for separated data subsets by a method that can deal with separation such as Firth’s penalization. Another, less frequent problem is the occurrence of bootstrap resamples or CV subsets with linearly dependent explanatory variables, e.g. if a binary explanatory variable is restricted to one category and thus is collinear with the constant. Such a variable would be omitted in a data analysis, but for the sake of simplicity, we just discarded those bootstrap resamples or CV subsets. Finally, the binary outcome might be restricted to one category either in the data subset where the model has to be fitted or in the data subset where the model performance measure is calculated. In both situations, we discarded the affected bootstrap resamples or CV subsets.


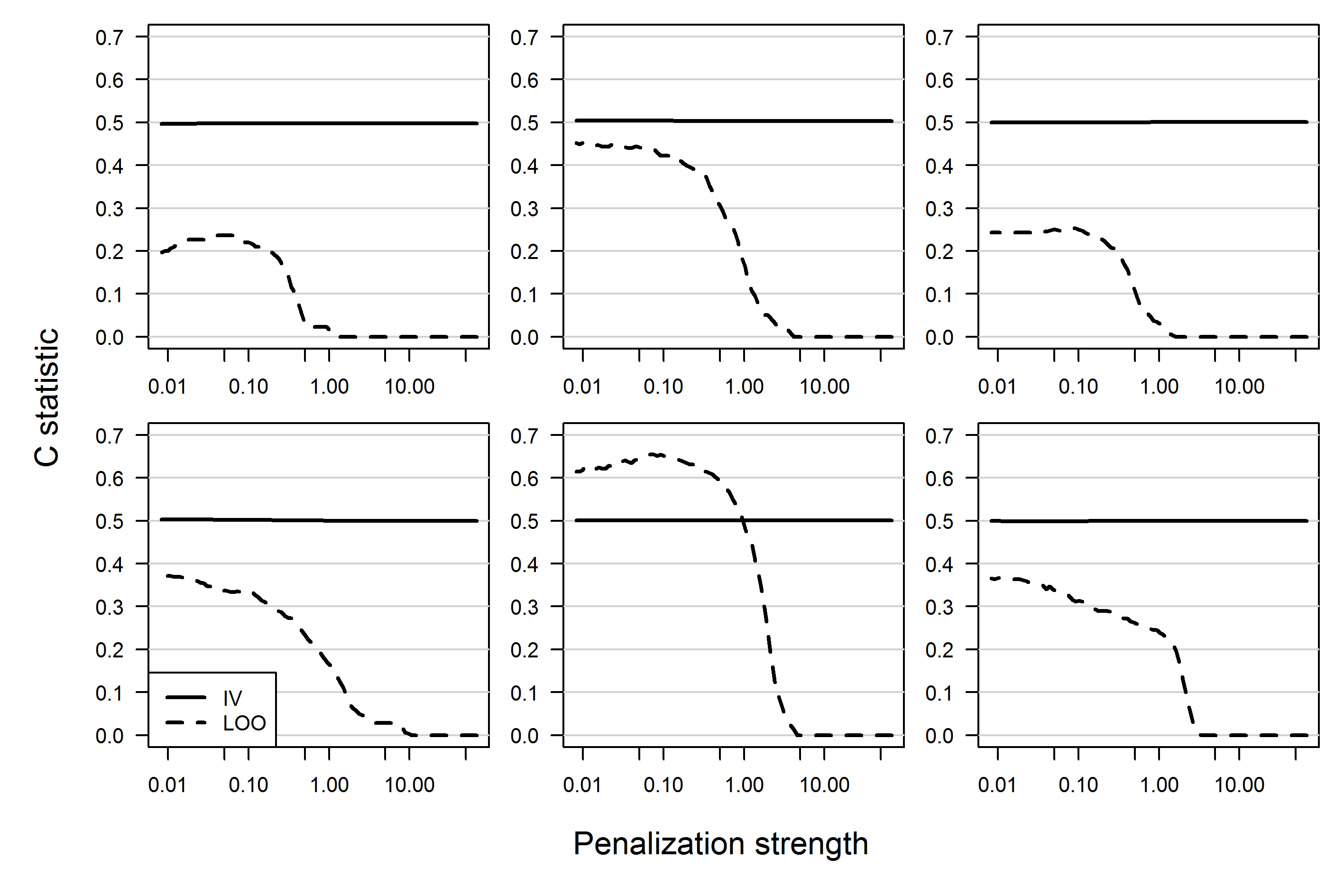


**Figure S1.** Independently validated (solid line) and leave-one-out crossvalidated (dashed line) c-statistics for different penalization strengths in ridge regression on six artificially constructed data sets. The data were created in the same way as for one of the scenarios in our simulation study (null scenario, sample size of 50, marginal event fraction of 0.25). The x-axis shows the tuning parameter in ridge regression (lambda in the R package glmnet) with higher values corresponding to stronger penalization. For each data set we fitted 96 ridge regression models corresponding to a series of log-equidistant tuning values. As in our simulation study, the independently validated c-statistics were obtained by validating the models on an independent data set consisting of 100,000 observations. As expected, the independently validated c-statistics are very close to the true value of 0.5.

LOO, leave-one-out crossvalidation; IV, independently validated.

S2. Data generating mechanism

Data generation was motivated by the structure of real data sets, where typically a mix of variables with different distributions is encountered. We generated one binary ($X_{1}$), one ordinal ($X_{2}$) and three continuous ($X_{3}$, $X_{4}$, $X_{5}$) explanatory variables as follows: first, we sampled five standard normal deviates $z_{i1}, \ldots, z_{i5}$ with correlation matrix as specified in Table S1. Next, we applied the transformations described in the Table S1 to obtain $x_{i1}, \ldots x_{i5}$. Finally, we winsorized the continuous variables at the values corresponding to their third quartile plus five times the interquartile distance in each simulated data set. Binary outcomes $y_{i}$ were drawn from Bernoulli distributions with the event probability following a logistic model, $P\left( Y \right|x_{i1}, \ldots x_{i5})=1/(1+exp(-\beta_{0}-\beta_{1}x_{i1}-\ldots-\beta_{5}x_{i5}))$. In this way, we obtained data sets with realistic distributions of explanatory variables. For instance, the continuous symmetric variable $X_{3}$ could represent age and the two right-skewed variables $X_{4}$ and $X_{5}$ could represent some lab parameters.

We considered twelve simulation scenarios in a factorial design combining sample size ($n\in\{50,100\}),$ marginal event fraction ($E\left( y \right)\in\{0.25, 0.5\}$) and effect size (strong or weak effects of all explanatory variables, or null scenarios with no effects). For each scenario we chose the intercept $\beta_{0}$ such that the desired marginal event fraction was approximately achieved. To simulate ‘strong effects’ scenarios, we set the model coefficients $\beta_{1}$ to 0.69 and $\beta_{2}$ to -0.345. For the continuous variables, we set $\beta_{3}$ to ‑0.0363, $\beta_{4}$ to 0.0031, and $\beta_{5}$ to ‑0.0039, corresponding to odds ratios of 2 or 1/2 when comparing the fifth and the first sextiles of the distribution functions of the corresponding explanatory variables. To simulate ‘weak effects’ we set $\beta_{1},\ldots,\beta_{5}$ to half of those values. Finally, the null scenarios were obtained by setting $\beta_{1},\ldots,\beta_{5}$ to 0. For each scenario we created 1 000 data sets.

**Table S1.** Construction of explanatory variables in the simulation study, following Binder H, Sauerbrei W, Royston P. Multivariable Model-Building with Continuous Covariates: 1. Performance Measures and Simulation Design. Germany: University of Freiburg; 2011. Square brackets $\left[ \ldots\right]$ indicate that the argument is truncated to the next integer towards $0$. The indicator function $\mathbf{1}_{\left\{ \ldots\right\}}$is equal to $1$ if the argument is true and $0$otherwise.

| **Underlying variable** | | **Correlation of underlying variables** | **Explanatory variable** | **Type** | **Correlation of explanatory variables** |
| --- | --- | --- | --- | --- | --- |
| $z_{i1}$ | $z_{i3} (0.8)$ | | $x_{i1}=\mathbf{1}_{\left\{ z_{i1}<0.6 \right\}}$ | binary | $x_{i3}(-0.6)$ |
| $z_{i2}$ | $z_{i4}\left( -0.5 \right), z_{i5} (-0.3)$ | | $x_{i2}=\mathbf{1}_{\left\{ z_{i2}\geq-1.2 \right\}}+\mathbf{1}_{\left\{ z_{i2}\geq0.75 \right\}}$ | ordinal | $x_{i4} \left( -0.4 \right), x_{i5}(-0.2)$ |
| $z_{i3}$ | $z_{i1} \left( 0.8 \right)$ | | $x_{i3}=[10 z_{i3}+55]$ | cont. | $x_{i1} (-0.6)$ |
| $z_{i4}$ | $z_{i2} \left( -0.5 \right), z_{i5}(0.5)$ | | $x_{i4}=[max(0,100\exp\left( z_{i4} \right)-20)]$ | cont. | $x_{i2}\left( -0.4 \right), x_{i5}(0.4)$ |
| $z_{i5}$ | $z_{i2} \left( -0.3 \right), z_{i4}(0.5)$ | | $x_{i5}=[\max\left( 0,80\exp\left( z_{i5} \right)-20 \right)]$ | cont. | $x_{i2}\left( -0.2 \right), x_{i4}(0.4)$ |

**Table S2.** Mean and standard deviation (x100) of independently validated (IV) c-statistics for different model estimators and all simulation scenarios. The standard deviation strongly depends on the number of new observations (in our case 100 000) used to estimate the IV c-statistics.

|  |  |  | **Mean IV c-statistic (x100)** | | | **Standard deviation of IV c-statistic (x100)** | | |
| --- | --- | --- | --- | --- | --- | --- | --- | --- |
| **Sample size** | **Event fraction** | **Effect size** | **ML** | **FL** | **RR** | **ML** | **FL** | **RR** |
| 100 | 0.25 | 0 | 49.99 | 49.99 | 49.99 | 0.21 | 0.21 | 0.21 |
|  |  | 0.5 | 56.87 | 56.83 | 57.15 | 3.43 | 3.44 | 3.6 |
|  |  | 1 | 67.59 | 67.54 | 67.78 | 2.7 | 2.73 | 2.64 |
|  | 0.5 | 0 | 50 | 50 | 50 | 0.18 | 0.18 | 0.18 |
|  |  | 0.5 | 57.72 | 57.72 | 58 | 2.98 | 2.98 | 3.03 |
|  |  | 1 | 68.32 | 68.32 | 68.44 | 2.13 | 2.13 | 2.07 |
| 50 | 0.25 | 0 | 50.01 | 50.01 | 50.01 | 0.21 | 0.21 | 0.21 |
|  |  | 0.5 | 55.38 | 55.26 | 55.63 | 4.14 | 4.21 | 4.48 |
|  |  | 1 | 64.87 | 64.77 | 65.35 | 4.74 | 4.85 | 4.74 |
|  | 0.5 | 0 | 50 | 50 | 50 | 0.19 | 0.19 | 0.19 |
|  |  | 0.5 | 55.78 | 55.76 | 56.04 | 3.85 | 3.86 | 4.1 |
|  |  | 1 | 65.78 | 65.76 | 66.24 | 3.92 | 3.93 | 3.63 |

For each simulated dataset and each model estimation method, the IV c-statistic was calculated from newly drawn data with 100,000 observations.

ML, maximum likelihood; FL, Firth’s logistic regression; RR, ridge regression.

**Table S3.** Mean and standard deviation (x100) of independently validated (IV) discrimination slope for different model estimators and all simulation scenarios. The standard deviation strongly depends on the number of new observations (in our case 100 000) used to estimate the IV discrimination slope.

|  |  |  | **Mean IV discrimination slope (x100)** | | | **Standard deviation of IV discrimination slope (x100)** | | |
| --- | --- | --- | --- | --- | --- | --- | --- | --- |
| **Sample size** | **Event fraction** | **Effect size** | **ML** | **FL** | **RR** | **ML** | **FL** | **RR** |
| 100 | 0.25 | 0 | -0.01 | -0.01 | 0 | 0.08 | 0.07 | 0.03 |
|  |  | 0.5 | 3.27 | 3.05 | 1.73 | 1.97 | 1.84 | 1.65 |
|  |  | 1 | 11.11 | 10.42 | 8.25 | 3.28 | 3.11 | 3.98 |
|  | 0.5 | 0 | 0 | 0 | 0 | 0.07 | 0.07 | 0.03 |
|  |  | 0.5 | 4.23 | 3.92 | 2.4 | 2.01 | 1.88 | 1.82 |
|  |  | 1 | 13.49 | 12.63 | 10.73 | 3.11 | 2.99 | 3.89 |
| 50 | 0.25 | 0 | 0 | 0 | 0 | 0.12 | 0.1 | 0.06 |
|  |  | 0.5 | 3.35 | 2.95 | 1.57 | 2.77 | 2.51 | 2.1 |
|  |  | 1 | 11.05 | 9.83 | 7.09 | 4.71 | 4.31 | 5.27 |
|  | 0.5 | 0 | 0 | 0 | 0 | 0.12 | 0.1 | 0.06 |
|  |  | 0.5 | 4.03 | 3.53 | 1.94 | 2.92 | 2.61 | 2.29 |
|  |  | 1 | 13.08 | 11.61 | 8.92 | 4.48 | 4.12 | 5.41 |

For each simulated dataset and each model estimation method, the IV discrimination slope was calculated from newly drawn data with 100,000 observations.

ML, maximum likelihood; FL, Firth’s logistic regression; RR, ridge regression.

**Table S4.** Mean and standard deviation (x100) of independently validated (IV) Brier score for different model estimators and all simulation scenarios. The standard deviation strongly depends on the number of new observations (in our case 100 000) used to estimate the IV Brier score.

|  |  |  | **Mean IV Brier score (x100)** | | | **Standard deviation of IV Brier score (x100)** | | |
| --- | --- | --- | --- | --- | --- | --- | --- | --- |
| **Sample size** | **Event fraction** | **Effect size** | **ML** | **FL** | **RR** | **ML** | **FL** | **RR** |
| 100 | 0.25 | 0 | 20.04 | 19.91 | 19.15 | 0.79 | 0.73 | 0.52 |
|  |  | 0.5 | 19.41 | 19.28 | 18.88 | 0.81 | 0.75 | 0.53 |
|  |  | 1 | 17.85 | 17.73 | 17.70 | 0.74 | 0.68 | 0.63 |
|  | 0.5 | 0 | 26.66 | 26.42 | 25.52 | 0.94 | 0.83 | 0.61 |
|  |  | 0.5 | 25.48 | 25.28 | 24.96 | 0.92 | 0.82 | 0.59 |
|  |  | 1 | 23.01 | 22.87 | 22.88 | 0.86 | 0.78 | 0.76 |
| 50 | 0.25 | 0 | 21.57 | 21.04 | 19.59 | 1.69 | 1.45 | 1.27 |
|  |  | 0.5 | 20.97 | 20.43 | 19.48 | 1.68 | 1.43 | 1.24 |
|  |  | 1 | 19.43 | 18.90 | 18.69 | 1.71 | 1.45 | 1.42 |
|  | 0.5 | 0 | 28.38 | 27.60 | 26.01 | 1.79 | 1.45 | 1.29 |
|  |  | 0.5 | 27.31 | 26.59 | 25.76 | 1.95 | 1.62 | 1.42 |
|  |  | 1 | 24.79 | 24.16 | 24.13 | 1.91 | 1.60 | 1.57 |

For each simulated dataset and each model estimation method, the IV Brier score was calculated from newly drawn data with 100,000 observations.

ML, maximum likelihood; FL, Firth’s logistic regression; RR, ridge regression.


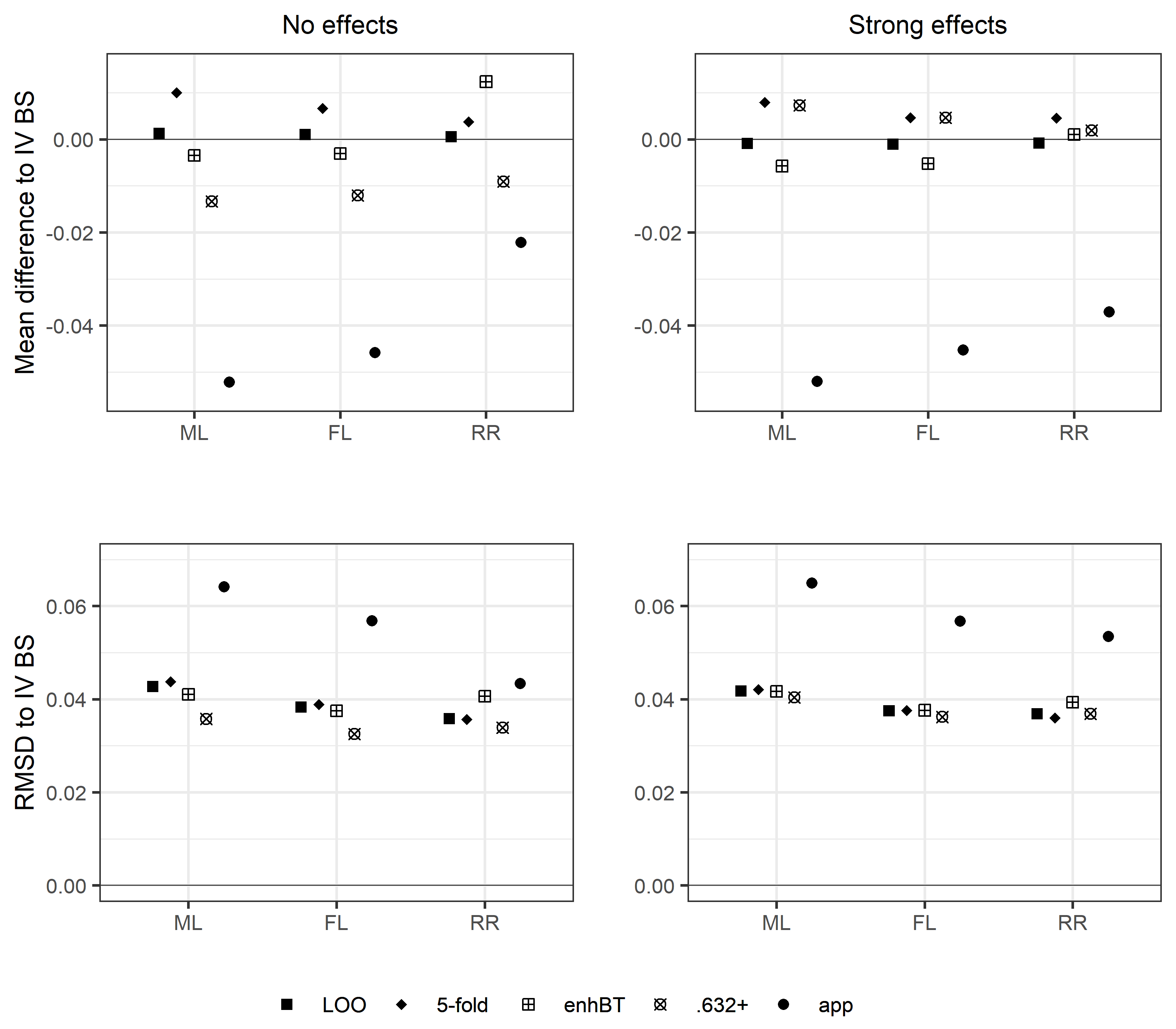


**Figure S2.** Mean and root mean squared differences (RMSD) between Brier scores (BS) computed by data resampling techniques and independently validated (IV) BS for three different model estimators for the simulation settings with 50 observations, an event fraction of 0.25 and either no or strong effects. The Monte Carlo standard errors of both, the mean difference and of the root mean squared difference (x100), were smaller than 0.2 for all scenarios.

ML, maximum likelihood; FL, Firth’s logistic regression; RR, ridge regression.

LOO, leave-one-out crossvalidation; 5-fold, 5-fold crossvalidation; enhBT, enhanced bootstrap; .632+, .632+ bootstrap; app, apparent estimate.

For each estimator and each resampling technique, the mean difference was calculated as $\frac{1}{1000}\sum_{s=1}^{1000} {(b}_{s}- B_{s})$, where $b_{s}$ denotes the Brier score calculated by the respective resampling technique and $B_{s}$ is the IV Brier score for the respective estimator for the $s$-th generated data set. Its Monte Carlo standard error was obtained as $\left( \frac{1}{999\cdot1000}{\sum_{s=1}^{1000} {((b}_{s}- B_{s})-(\bar{b_{s}}-\bar{B_{s}}) )}^{2} \right)^{1/2}$, where $\bar{b_{s}}$ and $\bar{B_{s}}$ are the mean resampled and IV Brier scores, respectively. The root mean squared difference was computed as $\left( \frac{1}{1000}\sum_{s=1}^{1000} {{(b}_{s}-B_{s})}^{2} \right)^{1/2}.$ We used the jackknife to obtain Monte Carlo standard errors of the root mean squared differences, as described in Koehler E, Brown E, Haneuse SJPA. On the Assessment of Monte Carlo Error in Simulation-Based Statistical Analyses. Am Stat. 2009;63(2):155-162.

**Table S5.** Percentage of separated data sets for the twelve simulation scenarios in the full data sets, in the data sets used for model fitting in leave-one-outcrossvalidation, leave-pair-out crossvalidation and 5-fold crossvalidation, respectively, and in the bootstrap data sets.

| **Sample size** | **Event fraction** | **Effect size** | **Full data** | **LOO** | **LPO** | **5-fold** | **BT** |
| --- | --- | --- | --- | --- | --- | --- | --- |
| 100 | 0.25 | 0 | 0.1 | 0.1 | 0.1 | 0.3 | 1.1 |
|  |  | 0.5 | 0.9 | 0.9 | 0.9 | 1.8 | 4.1 |
|  |  | 1 | 2.6 | 2.7 | 2.9 | 5.8 | 10.8 |
|  | 0.5 | 0 | 0.0 | 0.0 | 0.0 | 0.0 | 0.0 |
|  |  | 0.5 | 0.0 | 0.0 | 0.0 | 0.0 | 0.1 |
|  |  | 1 | 0.0 | 0.0 | 0.0 | 0.1 | 0.4 |
| 50 | 0.25 | 0 | 2.9 | 3.1 | 3.6 | 6.3 | 12.5 |
|  |  | 0.5 | 9.5 | 9.9 | 10.3 | 15.1 | 23.3 |
|  |  | 1 | 18.2 | 18.8 | 20.0 | 26.0 | 35.9 |
|  | 0.5 | 0 | 0.1 | 0.1 | 0.2 | 0.7 | 2.6 |
|  |  | 0.5 | 0.3 | 0.3 | 0.4 | 1.1 | 3.6 |
|  |  | 1 | 1.2 | 1.3 | 1.4 | 2.9 | 6.9 |

We checked for separation using Ioannis Kosmidis’ R package brglm2, version 0.1.8.

LOO, leave-one-out crossvalidation; LPO, leave-pair-out crossvalidation; 5-fold, 5-fold crossvalidation; BT, bootstrap.

**Table S6.** Mean difference and root mean squared difference (x100) between winsorized c-statistics computed by different resampling techniques and the independently validated (IV) value (as presented in Table S2) for simulation scenarios with sample size of 50 and event fraction of 0.25. Resampled c-statistics were winsorized by replacing values smaller than 0.5 by 0.5. Figure 3 shows the analogous results for the untransformed c-statistics.

|  |  | **Mean difference (x100)** | | | | | | **Root mean squared difference (x100)** | | | | | |
| --- | --- | --- | --- | --- | --- | --- | --- | --- | --- | --- | --- | --- | --- |
| **Effect size** | **Estimator** | **LOO** | **LPO** | **5-fold** | **enhBT** | **.632+** | **app** | **LOO** | **LPO** | **5-fold** | **enhBT** | **.632+** | **app** |
| 0 | ML | 2.76 | 5.53 | 4.99 | 8.08 | 5.08 | 20.20 | 5.98 | 9.21 | 8.53 | 11.26 | 8.89 | 21.48 |
|  | FL | 2.87 | 5.61 | 5.08 | 8.10 | 5.10 | 20.06 | 6.29 | 9.29 | 8.63 | 11.30 | 8.91 | 21.34 |
|  | RR | 1.45 | 5.68 | 5.13 | 7.25 | 5.05 | 18.47 | 4.73 | 9.34 | 8.74 | 10.67 | 8.89 | 19.91 |
| 0.5 | ML | -0.07 | 3.36 | 2.68 | 6.02 | 3.08 | 17.50 | 7.91 | 9.68 | 9.17 | 10.83 | 9.57 | 19.12 |
|  | FL | 0.02 | 3.40 | 2.65 | 6.00 | 2.87 | 17.46 | 8.01 | 9.68 | 9.15 | 10.80 | 9.45 | 19.08 |
|  | RR | -2.07 | 3.32 | 2.60 | 5.00 | 2.72 | 15.71 | 8.21 | 9.81 | 9.24 | 10.51 | 9.56 | 17.69 |
| 1 | ML | -3.51 | 0.96 | 0.01 | 3.30 | 0.61 | 13.17 | 10.34 | 10.21 | 9.99 | 10.20 | 10.36 | 15.35 |
|  | FL | -3.26 | 0.98 | -0.02 | 3.22 | 0.33 | 13.12 | 10.40 | 10.21 | 9.98 | 10.18 | 10.31 | 15.32 |
|  | RR | -6.02 | 0.70 | -0.17 | 2.40 | 0.21 | 11.69 | 11.92 | 10.17 | 9.92 | 10.28 | 10.35 | 14.38 |

ML, maximum likelihood; FL, Firth’s logistic regression; RR, ridge regression.

LOO, leave-one-out crossvalidation; LPO, leave-pair-out crossvalidation; 5-fold, 5-fold crossvalidation; enhBT, enhanced bootstrap; .632+, .632+ bootstrap; app, apparent estimate.

For each estimator and each resampling technique, the mean difference was calculated as $\frac{1}{1000}\sum_{s=1}^{1000} {(c}_{s}- C_{s})$, where $c_{s}$ denotes the winsorized c-statistic calculated by the respective resampling technique and $C_{s}$ is the IV c-statistic for the respective estimator for the $s$-th generated data set. The root mean squared difference was computed as $\left( \frac{1}{1000}\sum_{s=1}^{1000} {{(c}_{s}-C_{s})}^{2} \right)^{1/2}.$

S3. A side remark on the simple bootstrap: resampling may increase the optimism

With the simple bootstrap, the parameter estimates from models fitted on bootstrap resamples (sampling $n$ observations with replacement from the original data) are used to calculate the c-statistic for the original data sample, see *Efron B, Tibshirani RJ. An Introduction to the Bootstrap: Chapman & Hall; 1993*. Usually this is repeated, say, 200 times and the estimates are averaged. The simple bootstrap is known to perform poorly compared to the more refined bootstrap techniques which we also considered in our study, see *Harrell FE. Regression Modeling Strategies: Springer; 2001*.

We have not included the simple bootstrap in the main presentation of our simulation results due to its known inferiority. Though, some results are worth to report. In all simulation scenarios, the simple bootstrap gave median discrimination slopes even more optimistic than the apparent ones. In other words, the simple bootstrap increased the optimism instead of correcting it as we would expect. This phenomenon was observed with each model estimator. At first glance, it might appear counterintuitive that models fitted on bootstrap resamples discriminate the original outcomes better than the model fitted on the original data, but there is a simple explanation: models fitted on bootstrap resamples with their repeated observations tend to give more extreme estimated probabilities than the model fitted on the original data.

The c-statistics and Brier scores estimated by the simple bootstrap were also severely overoptimistic but on average smaller than their apparent counterparts.
